# Automatic Detection of Hard Exudates in Color Retinal Images Using Dynamic Threshold and SVM Classification: Algorithm Development and Evaluation

**DOI:** 10.1155/2019/3926930

**Published:** 2019-01-23

**Authors:** Shengchun Long, Xiaoxiao Huang, Zhiqing Chen, Shahina Pardhan, Dingchang Zheng

**Affiliations:** ^1^College of Computer Science and Technology, Zhejiang University of Technology, Hangzhou 310023, China; ^2^Eye Center, the Second Affiliated Hospital of Zhejiang University School of Medicine, Hangzhou 310000, China; ^3^Vision and Eye Research Unit (VERU), School of Medicine, Anglia Ruskin University, Chelmsford, UK; ^4^Department of Medical Science and Public Health, Faculty of Medical Science, Anglia Ruskin University, Chelmsford, UK

## Abstract

Diabetic retinopathy (DR) is one of the most common causes of visual impairment. Automatic detection of hard exudates (HE) from retinal photographs is an important step for detection of DR. However, most of existing algorithms for HE detection are complex and inefficient. We have developed and evaluated an automatic retinal image processing algorithm for HE detection using dynamic threshold and fuzzy C-means clustering (FCM) followed by support vector machine (SVM) for classification. The proposed algorithm consisted of four main stages: (i) imaging preprocessing; (ii) localization of optic disc (OD); (iii) determination of candidate HE using dynamic threshold in combination with global threshold based on FCM; and (iv) extraction of eight texture features from the candidate HE region, which were then fed into an SVM classifier for automatic HE classification. The proposed algorithm was trained and cross-validated (*10* fold) on a publicly available e-ophtha EX database (*47* images) on pixel-level, achieving the overall average sensitivity, PPV, and F-score of* 76.5%*,* 82.7%,* and* 76.7%*. It was tested on another independent DIARETDB1 database (*89* images) with the overall average sensitivity, specificity, and accuracy of* 97.5%*,* 97.8%,* and* 97.7%*, respectively. In summary, the satisfactory evaluation results on both retinal imaging databases demonstrated the effectiveness of our proposed algorithm for automatic HE detection, by using dynamic threshold and FCM followed by an SVM for classification.

## 1. Introduction

Diabetic retinopathy (DR) is one of the major complications of diabetes that can lead to vision loss. The prevalence of DR is expected to grow exponentially, and the global population of DR patients is expected to increase to* 191.0* million by 2030 [1]. The severity of DR is categorized according to the number of microaneurysms, hemorrhages, exudates, and neovascularization. The progress of DR is normally classified into normal retina, background DR, nonproliferative DR (NPDR), proliferative DR (PDR), and/or macular edema (ME) [2]. Regular screening to detect retinopathy can potentially reduce the risk of blindness of patients.

**Figure 1:**
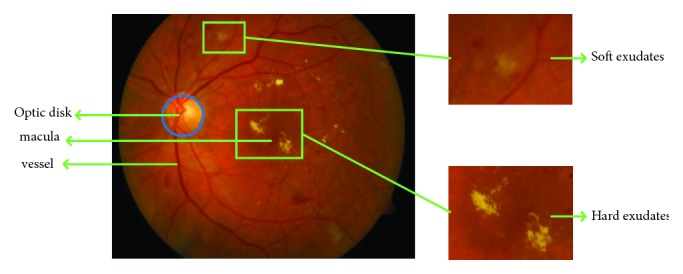
Example of retinal fundus image with exudates regions (zoom into the soft exudates region and hard exudates region).

It is known that the occurrence of hard exudates (HE) is one of the main threats to vision loss especially when they occur near or on fovea [3].  Figure 1 shows an example of color retinal fundus image with HE. HE appears at late background and NPDR stages on the surface of retina as bright yellowish or white at different locations [4] and with variable shapes and sizes ranging from a few pixels to thousands of pixels in the retinal images. It is well accepted that the detection of HE in color retinal images plays a vital role in DR diagnosis and monitoring the progress of treatment. HE detection is therefore the main emphasis of this study.

HE is usually visually graded which is time-consuming and susceptible to observer errors [5]. The computer-aided detection of HE would potentially assist in achieving fast and accurate diagnosis. Many published algorithms have been developed for automatic HE detection in retinal images using four main strategies: thresholding, edge detection, region growing, and classification. Using the global threshold method and edge detection to achieve exudates detection automatically and accurately is very challenging due to the uneven intensity of the exudates, and the low contrast between exudates and retinal background [6]. Liu et al. [7] proposed a semiautomatic approach to detect low intensity exudates using local thresholds, which required the operator to select the local threshold manually based on the histogram of subimages. Region growing has also been implemented [8] to detect the exudates, which suffers from the difficulties of selecting the seed point and stopping criteria in region growing due to the wide variety of color distribution and nonhomogeneous illumination. Recently, different classification methods for exudates detection have been proposed to achieve fully automatic detection. An SVM classifier in combination with a Gaussian scale space approach has been used to differentiate between soft exudates, HE and outliers [9]. Other classification methods, including the bootstrapped decision trees [10], a Naive–Bayes classifier optimized further by an adaptive boosting technique [11], and random forest method [12], have also been used. Unfortunately, the classification results from applying the above methods for HE detection are not clinically satisfactory enough due to various qualities of retinal images. This requires a more effective image segmentation method before classification.

Due to the large variety of the exudates in size, intensity, shape, and contrast, and the noise or artifacts during the image acquisition process, segmenting the small proportion of exudates pixels from the whole retinal images is challenging, leading to unsatisfactory detection accuracy for clinical applications. For general color image segmentation, fuzzy C-means (FCM), an unsupervised fuzzy clustering, has been widely used [13], where the global threshold is commonly used. However, using a global threshold may ignore the local details of the image. Dynamic threshold has been used, but this is more prone to shadow and man-made boundaries. It has been approved by Moghaddam and Cheriet [14] that using dynamic threshold in combination with the global threshold can significantly improve the effectiveness of segmentation of areas of interest in other fields, such as melasma image segmentation and cell cluster segmentation for in situ microscopy [15]. To date, the application of employing dynamic threshold in combination with global threshold based on FCM has not been attempted in retinal image segmentation. In this study, we use this combined approach for determining candidates of HE from retinal images. After image segmentation, the segmented regions are normally classified into two disjoint classes using a neural network or support vector machines (SVM). Literature suggests that SVM is more practical than neural networks for small size of training data [16]. In machine learning, SVM is a supervised learning model with associated learning algorithms that analyze data for classification and regression analysis. The SVM is characterized by the ability to simultaneously minimize empirical errors and maximize the geometric edge region [17]. SVM is therefore implemented in this study.

The aim of this study was therefore to develop and evaluate a HE detection algorithm using dynamic threshold and FCM in combination with SVM. Specifically, after OD localization based on the image gray-scale value and retinal blood vessels distribution, the dynamic threshold matrix will be obtained using FCM in each subimage, which will be combined with global threshold matrix to obtain the exudates candidate regions, and followed with SVM classification to achieve automatic HE detection.

## 2. Methods

### 2.1. Retinal Image Databases

Our proposed algorithm was developed and tested on two publicly available databases of retinal images (the DIARETDB1 [18] and the e-ophtha EX [12]). DIARETDB1 database contains* 89* color fundus images with 50° Field of View (FOV) and the size of* 1500 *×* 1152* pixels, of which only* 5* are normal; others contain different lesions. In this database, the different regions with HE have been manually labelled by four specialists from each image to determine whether a retinal image contains exudates. Human graders marked* 571* regions as exudates DIARETDB1. The e-ophtha EX dataset contains* 47* images with exudates regions and* 35* exudates-free images, where only the* 47* images with exudates regions were used in this study. Since the ground truth in DIARETDB1 is based on image-level and the e-ophtha EX is the only publicly available database which has provided pixel-level annotation for exudates segmentation, the e-ophtha EX was selected to train and cross-validate our algorithm on pixel-level, and the DIARETDB1 was used for additional independent test to discriminate whether a retinal image contains exudates.

### 2.2. Algorithm Development for Automatic HE Detection

**Figure 2:**
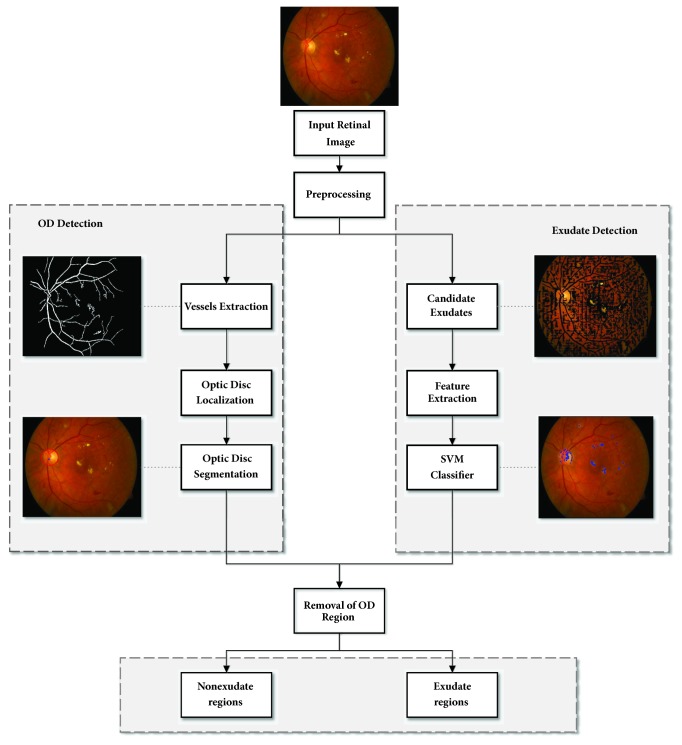
Flow chart of our proposed algorithm for automatic detection of HE.

As shown in Figure 2, our proposed algorithm for HE detection was composed of four main stages: (i) image preprocessing, (ii) OD localization, (iii) exudates candidate regions determination, and (iv) HE features extraction and classification. Matlab (2016a) was used in the environment of 64 bit Windows 10 operating system with 2.9 GHz Intel Core i5 CPU and 16GB memory.

#### 2.2.1. Retinal Imaging Preprocessing

The preprocessing stage is crucial due to the intrinsic characteristics of retinal images. Retinal images often have poor and varying contrasts due to many factors including the noises introduced during the imaging acquisition process and the improper reflection of camera flash and retinal pigmentation. Additionally, the uneven illumination increases the intensity level near OD and decreases in regions away from OD. All these factors have significant impact on HE detection.

In our algorithm, color intensity normalization and contrast enhancement of the fundus photographs were operated with the size of retinal image rescaled to* 512 *×* 512* pixels. As proposed by Clara et al. [19], color normalization was performed by enhancing luminance plane of YIQ color model instead of enhancing each color plane of RGB. The modified process is as follows:

(1) $$\begin{array}{c} Y_{mod}=aY-bI-cQ \end{array}$$

**Figure 3:**
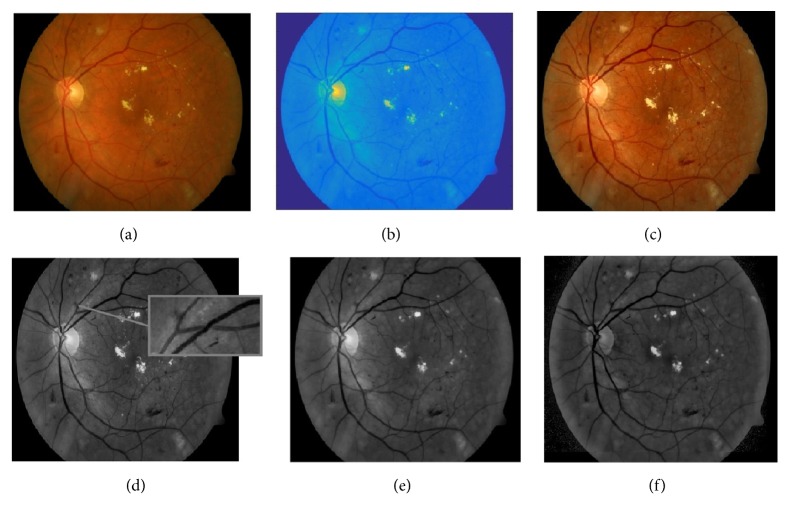
Example of retinal fundus image preprocessing. (a) Original retinal image. (b) Color normalized YIQ plane image. (c) Enhanced RGB plane image. (d) Green channel image after CLAHE (zoom into the blood vessels with brighter strip). (e) Green channel image after morphological opening. (f) Mean filtering of (e).

The modified color model YIQ was then converted back to RGB color model, as shown in the first three images in Figure 3. The empirical values of* 1.8*,* 0.9,* and* 0.9* were used for parameters a, b, and c, respectively, with which satisfactory results were achieved when the images were converted back to RGB color model, producing greater contrast between the HE and the background for the next step of HE detection.

It has been observed that the OD appears most contrasted in the green channel when compared to red and blue channels in the RGB retinal images [20]. Additionally, as the red channel is too saturated and the blue channel is the darkest color channel that does not contain much information, the green channel image was only used for the HE detection. Furthermore, in order to remove some bright strips down the central length of the blood vessels, the green plane of the image after contrast limited adaptive histogram equalization (CLAHE) was filtered by applying a morphological opening using a three-pixel diameter disc [21]. Next, the illumination equalization method in [22] was used to correct shade as follows:

(2) $$\begin{array}{c} I_{ie}=I-I_{bg}+u \end{array}$$

where a mean filter of size* 51 *×* 51* was applied to the green channel image* I* to generate a background image *I*_*bg*_ which was then subtracted from the* I* to correct for shade variations. Finally, the average intensity* u* of green channel image* I* was added to keep the gray range same as in the* I*. The example images during the process are shown in Figure 3.

#### 2.2.2. Optic Disc Detection and Masking

OD localization is an essential stage in our proposed algorithm because OD has similar properties as exudates in terms of color and brightness. The OD is a bright yellow disc in the retina where retinal blood vessels emerge. Therefore, the disc should be masked from the fundus image before further HE detection.

OD localization is relatively simple and fast in normal retinal images because it is where the largest cluster of brightest pixels is; however, this becomes more challenging in the images where the area of bright lesions is also large or OD is obscured by retinal blood vessels, for example, when there is a large hemorrhage on the disc [6]. In our proposed algorithm, the information of image brightness and retinal vasculature features were used for OD localization [23], which involved three steps: retinal blood vessels extraction, the center of OD localization, and OD segmentation.

**Figure 4:**
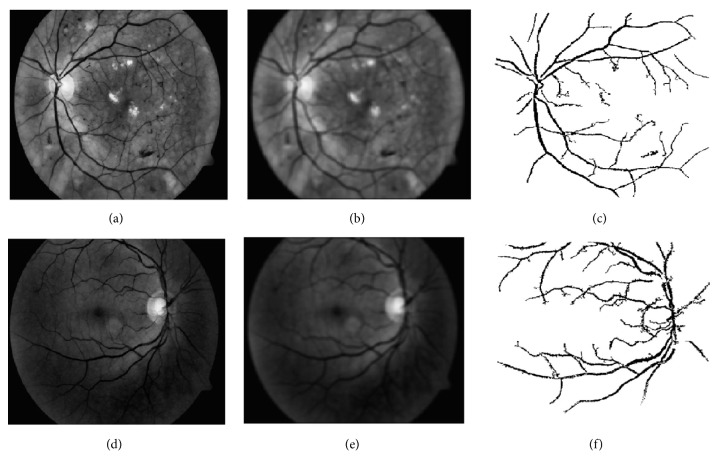
Examples of retinal blood vessels extraction on two retinal images with different illumination conditions. (a)+(d) Green channel images after CLAHE. (b)+(e) Mean filtering of (a) and (d) respectively. (c)+(f) Extracted retinal blood vessels.

*Retinal Blood Vessels Extraction*. In general, retinal blood vessels in the green channel fundus images do not have enough contrast in comparison with the surrounding background. An enhancement method of CLAHE [24] was applied to solve this problem. Next, a mean filtering with a* 9 *×* 9* pixel-kernel was used to blur the image to reduce the noises. The retinal blood vessels image *I*_*bv*′_ was obtained by subtracting the blurred image from the enhanced image by CLAHE, and the retinal blood vessels image *I*_*BV*_ was obtained by thresholding operator [25] applied to *I*_*bv*′_. This process is shown in Figure 4, where two example images with different illumination conditions are given.

**Figure 5:**
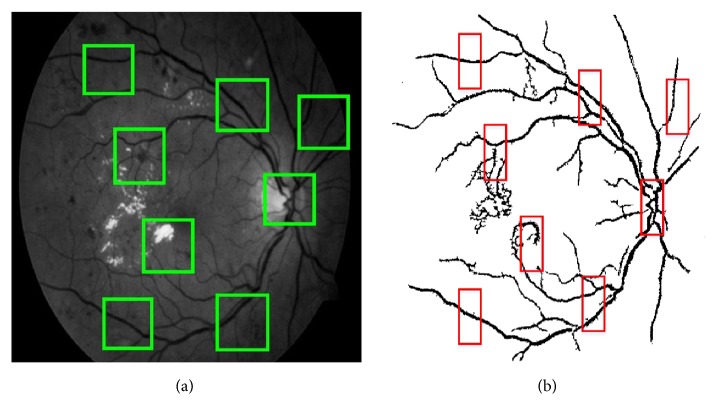
Illustration of the process for localizing the center of the optic disc. (a) Green channel image. The green boxes indicate the size of the mean filter. (b) The red boxes show the size of the window used to calculate the local average intensity of *I*_*BV*_.

*The Center of Optic Disc Localization*. Retinal blood vessels originate from OD and spread outwards to the retina and the macular region. The vessels are generally aligned vertically in the vicinity of OD [26]. In order to obtain retinal blood vessels position information, a mean filter of size* 61 *×* 61* was applied to the green channel image* I* to generate an average intensity image *I*_*G*′_, and the *I*_*BV*′_ (local average intensity of *I*_*BV*_) was computed from the average intensity of the pixels within an* N *×* M* window as illustrated in Figure 5. In this study, the window size* N* was between* 50* and* 60* pixels, and* M* was between* 20* and* 25* pixels. Next, in order to combine the brightness features and blood vessels position information from the green channel image, each pixel *I*_*OD*_(*r*, *c*) in the image was adjusted as follows:

(3) $$\begin{array}{c} I_{OD}\left(\;r,c\;\right)=I_{BV^{\prime }}\left(\;r,c\;\right)-1.2*I_{G^{\prime }}\left(\;r,c\;\right) \end{array}$$

**Figure 6:**
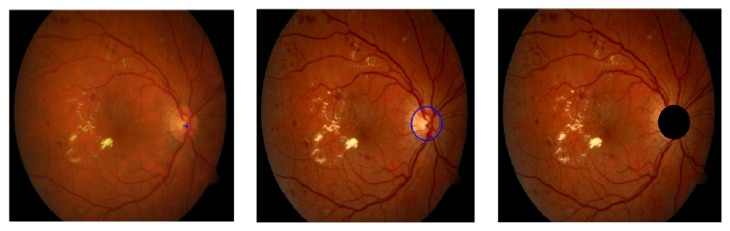
Demonstration of optic disc (OD) localization, segmentation, and masking. (a) Localization of OD center. (b) Segmentation of OD. (c) Masking OD.

The image *I*_*OD*_ was then traversed with the minimum point identified as the center of OD, as shown in Figure 6(a).

*Optic Disc Segmentation*. To detect the OD boundary, the size* m *×* n* of region of interest (ROI) was defined based on the localization result of OD center, where* m* and* n* were one-ninth of the respective dimensions of the image multiplied. Since the OD in the retinal images has circular boundary shape [27], a circular Hough transform was applied to segment the OD boundary [23, 28, 29]. The Hough transform is a widely considered technique in Computer Vision and Pattern Recognition to detect geometrical features that can be defined through parametric equations like straight lines and circles. The OD segmentation by applying Hough transform is shown in Figure 6(b). Lastly, the segmented OD was masked to avoid the interference to the following HE detection, as shown in Figure 6(c).

#### 2.2.3. Detection of Hard Exudates

There were two main procedures. FCM clustering was firstly used to get the local dynamic threshold of each subimage, which was then combined with global threshold matrix to segment color retinal images. Next, an SVM classification was applied to distinguish exudates and nonexudates regions.

*Retinal Image Segmentation Using FCM*. The following describes the image segmentation process using the dynamic threshold in combination with global threshold based on FCM clustering:

(1) The retinal image was divided into a series of subimages (*K* subimages), and FCM algorithm was used to assign pixels in each subimage to different categories by using fuzzy memberships. FCM is an iterative optimization that minimized the cost function defined as follows:

(4) $$\begin{array}{c} J\left(\;U,V\;\right)=\overset{n}{\underset{i=1}{\sum }}\;\overset{c}{\underset{k=1}{\sum }}{\left(\;u_{ki}\;\right)}^{m}{\left\|\;x_{i}-v_{k}\;\right\|}^{2} \end{array}$$

where *u*_*ki*_ represents the membership of pixel *x*_*i*_ in the* k*th cluster and *v*_*k*_ represents the clustering center of the* k*th cluster. Considering that the gray-scale value was used as the only feature for clustering, the midpoint of the clustering center line was used as the threshold in the segmentation sense, where the mean of the two clustering centers was obtained as the threshold of the subimage;

(2) The entire original retinal image pixels were classified in a similar way as above to obtain the global threshold and construct the global matrix* S* with the same size as the original image.

(3) After the interpolation of the thresholds of the respective subimages into a dynamic threshold matrix* D* of the same size as the entire original image, a mean filter of size* 10 *×* 10* was applied to the matrix* D*.

(4) The final threshold matrix* T* was constructed as

(5) $$\begin{array}{c} T=kS+\left(\;1-k\;\right)D \end{array}$$

where the value of* k* was set to 0.1.

(5) The segmentation result was obtained by comparing the threshold matrix* T* with the retinal image.

**Figure 7:**
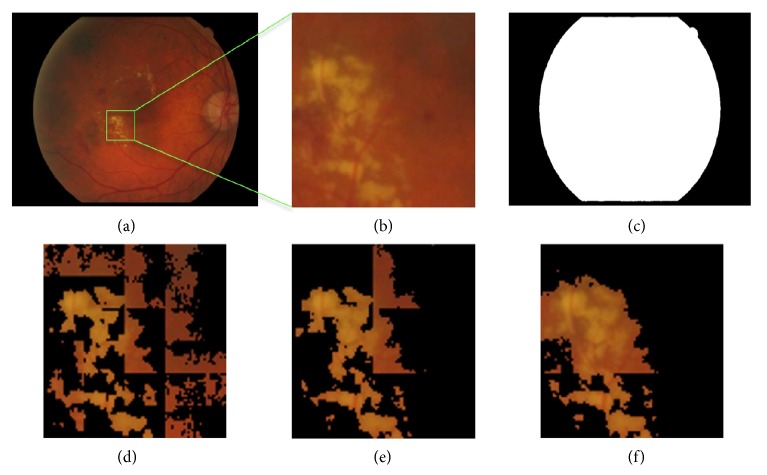
FCM clustering segmentation with different subimage sizes. (a) Original retinal image. (b) Zooming into the exudates region. (c) Segmentation result of original image using FCM. (d) Segmentation result with the subimage size of* 15*×*20* pixels. (e) With the subimage size of* 30*×*40* pixels. (f) With the subimage size of* 60*×*80* pixels. Note: the background is filled with black.

The size of the subimage affects the retinal imaging segmentation results.  Figure 7 shows the FCM clustering results for different subimage sizes. Taking both the running time and accuracy of local threshold into consideration, the size of* 30*×*40* pixels was selected as the most suitable subimages size.

*Feature Extraction for Hard Exudates Detection*. In order to further segment the exudates regions from the exudates candidates, some significant features that were commonly used by eye care practitioners to visually distinguish HE from other types of lesions were extracted from each region and used as inputs of SVM. The key features included the following:

- Mean green channel intensity (f1): a mean filter of size* 3*×*3* was applied to the green channel image. This feature indicates the gray-scale intensity for all pixels. Again, only the features from the green channel were extracted.
- Gray intensity (f2): it was the gray-scale value of each pixel.
- Mean hue (f3), mean saturation (f4), and mean value (f5) of retinal image in HSV color model: a mean filter of size* 3*×*3* was, respectively, applied to the three channel image *I*_*h*_, *I*_*s*_, *I*_*v*_. Because exudates are the bright lesions on the surface of retina, the information about saturation and brightness (f4 and f5) of retinal image is also important.
- Energy (f6): energy was the sum of intensity squares of all pixel values in eight-convexity.
- Standard deviation (SD) of the green channel image (f7): the morphological opening operation was applied to the green channel image to preserve foreground regions that have a similar shape to the structuring element or that completely contain the structuring element, while eliminating all the other regions of foreground pixels.
- Mean gradient magnitude (f8): it was the magnitude of the directional change in intensity of edge pixels. It helps in distinguishing strong and blurry edges to differentiate between exudates and other bright lesions [3].

In comparison with other published algorithms where dozens of features were used [3, 9, 11], only eight key features were extracted in this study to reduce processing time while maintaining the accuracy of HE extraction.

**Figure 8:**
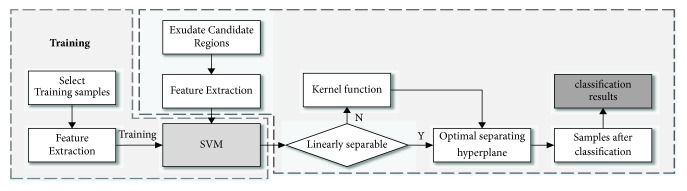
Flow chart of the SVM classification process.

*SVM Classification*. The flow chart of the SVM classification algorithm is shown in Figure 8. Briefly, the features extracted from the test images were fed into the trained SVM classifier to output a binary matrix representing the classification results. In this study, SVM was applied along with kernel function based on radial basis function (RBF). RBF kernel function has been widely used with two parameters (C and *γ*) obtained from the grid search method.

For training and cross-validation purposes, a few small regions (each image is about 1-10 regions, size between* 50* and* 250* pixels) of each of the* 47* ground truth images were manually selected from the e-ophtha EX dataset as training samples. These selected regions have been divided into exudates regions and nonexudates regions. Using the e-ophtha EX dataset, a* 10*-fold cross-validation was applied to evaluate the ability of SVM classifier on pixel-level. The database was randomly split into* 10* mutually exclusive subsets (the folds) *D*_1_, *D*_2_, *D*_3_,…, *D*_10_, approximately of equal size. The classifier was trained on* 42* selected training images and tested the remaining* 5* images to output a binary matrix representing the classification result. This procedure was repeated* 10* times.

For each training image, a certain number of pixels (ranges from* 50* to* 250*) were manually selected to construct training vector set. Each pixel constituted a feature vector from the eight key features. *x*_*i*_ represents the input sample feature vector set as follows:

(6) $$\begin{array}{c} x_{i}=\left(\;f1,f2,f3,\ldots ,f8\;\right) \end{array}$$

The acquired training sample set (*x*_*j*_, *y*_*j*_) was input to train the SVM. *y*_*j*_ is the category flag:

(7) $$\begin{array}{c} y_{j}=\left\{\begin{array}{cc} -1, & x_{j}\in A\\ 1, & x_{j}\in B \end{array}\right. \end{array}$$

*j* ⊂ {1,2,…,…, *W*},* W* is the dimension of the set of sample feature vectors.* A* and* B,* respectively, represent the HE and non-HE regions. In this study, around* 7200* training vectors (or pixels) from the* 42* training images were manually selected by an operator (*W=7200*).

The* 10*-fold cross-validation procedure was repeated five times by five different operators to manually select a region from each training image and then run the above procedure to evaluate the algorithm reliability.

### 2.3. Ensemble Evaluation Criteria

The evaluation criteria for HE identification were presented at two levels: pixel-level and image-level depending on which database was used. The pixel-level determination was based on whether each pixel of the classification result from the e-ophtha EX dataset has exudates in comparison with precisely labelled ground truth. The image-level HE detection was based on the presence or absence of HE in the classification result to determine whether a retinal image in the DIARETDB1 contains exudates.

#### 2.3.1. Pixel-Level Evaluation on e-Ophtha EX Database

The evaluation can be classically performed by counting the number of pixels which were correctly classified. However, this approach was inappropriate for exudates segmentation evaluation because the contours of exudates do not match perfectly between the determinations from different observers, resulting in weak agreement on exudates determination. In this study, a hybrid validation method was used, where a minimal overlap ratio between ground truth and candidates was required.

Given the segmented exudates connected component set {*D*_1_, *D*_2_,…, *D*_*N*_} and the ground truth exudates component set {*G*_1_, *G*_2_,…, *G*_*M*_}, we have the following.

A pixel was considered as a true positive (TP) if it belongs to

(8) D∩G∪Di ∣ Di∩GDi>σ∪Gj ∣ Gj∩DGj>σ

where |·| is the cardinal of a set and *σ* is a parameter ranging from* 0* to* 1*. *σ* was set to* 0.2* as used by Zhang et al. [12].

A pixel was considered as a false positive (FP) if it belongs to

(9) $$\begin{array}{c} \left\{\;D_{i}\;|\;D_{i}\cap G=⌀\;\right\}\cup \left\{\;D_{i}\cap \bar{G}\;|\;\frac{\left|D_{i}\cap G\right|}{\left|D_{i}\right|}\leq \sigma \;\right\} \end{array}$$

or as a false negative (FN) pixel if it belongs to

(10) $$\begin{array}{c} \left\{\;G_{j}\;|\;G_{j}\cap D=⌀\;\right\}\cup \left\{\;G_{j}\cap \bar{D}\;|\;\frac{\left|G_{j}\cap D\right|}{\left|G_{j}\right|}\leq \sigma \;\right\} \end{array}$$

The remaining pixels were considered as true negative (TN) pixels.

In this study, the four classes were clearly unbalanced as TP, FN, and FP were negligible in practice with respect to TN, computing the specificity, i.e.,* TN/(FP+TN)*, and a receiver operating characteristic (ROC) curve, which is not appropriate. Sensitivity (*S* = T*P*/(*TP* + *FN*)), positive prediction value (*PPV* = *TP*/(*TP* + *FP*)), and F-score ((2 × *S* × *PPV*)/(*S* + *PPV*)) were therefore used as the performance of HE detection. The PPV combined both TP and FP, indicating the ratio of detected exudates pixels annotated as exudates pixels by specialists.

#### 2.3.2. Image-Level Evaluation on DIARETDB1 Database

**Figure 9:**
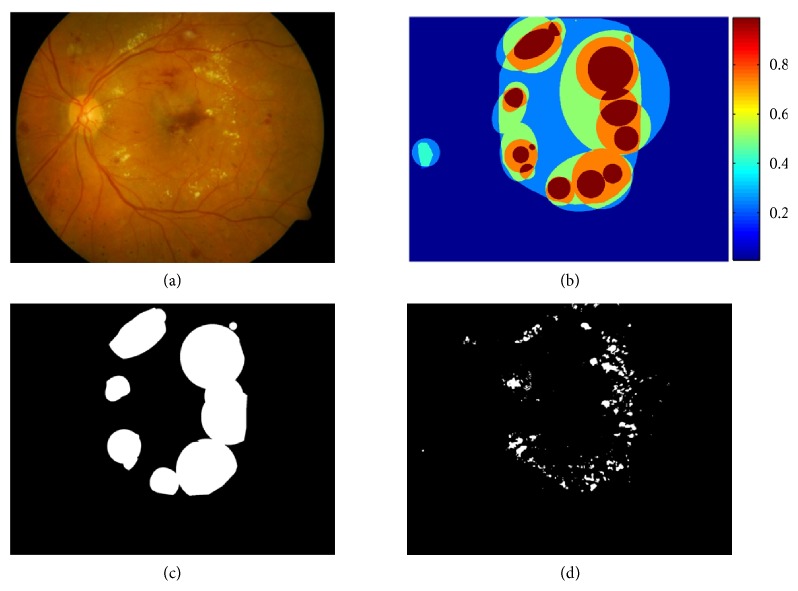
Example of one retinal image in DIARETDB1 database. (a) Original image. (b) Exudates regions labelled by four specialists (color decodes the ground truth confidence). (c) The exudates regions where the ground truth confidence level ≥75%. (d) Segmentation result of our algorithm.

From clinical point of view, it would also be useful to evaluate the presence of exudates at the image-level, especially for DR screening applications. In order to evaluate the robustness of our algorithm, our algorithm was independently tested to determine whether the testing image contains exudates using the* 89* images in the DIARETDB1 database, which has been labelled with ground truth at the image-level. As shown in Figure 9, each image was labelled by four specialists, if the ground truth confidence level is greater than or equal to* 75%*, the image was diagnosed with HE. At the image-level, if the image according to our algorithm and the ground truth both contain exudates region, the classification result for this retinal image was concluded as a TP. Matlab functionality for computing performance measures is publicly available at the DIARETDB1 web page [18]. For example, the processed image as Figure 9(d) was fed as an input into the evaluation protocol to obtain the evaluation outcomes (TP, TN, FP, FN). Three different evaluation parameters, including the sensitivity, specificity, and accuracy, were then used to determine the overall performance of HE detection. Their calculation formulas are shown as follows:

(11) Accuracy=TN+TPTP+FP+TN+FNSpecificity=TNTN+FPSentivity=TPTP+FN

### 2.4. Data Statistical Analysis

For the* 10*-fold cross-validation using the e-ophtha EX database, the sensitivity, PPV, and F-score were calculated for each image, with their mean and standard deviation (SD) across all the images calculated. Their SD between the five repeats performed by the five different operators were also calculated to demonstrate the reliability of our algorithm. ANOVA analysis was then performed to check the repeatability between the five repeats. For the independent test on the DIARETDB1 database, the overall mean sensitivity, specificity, and accuracy were calculated from all the* 89* images, which were simply compared with other published results using the same database.

## 3. Results

### 3.1. 10-Fold Cross-Validation Results on the e-Ophtha EX Database

**Figure 10:**
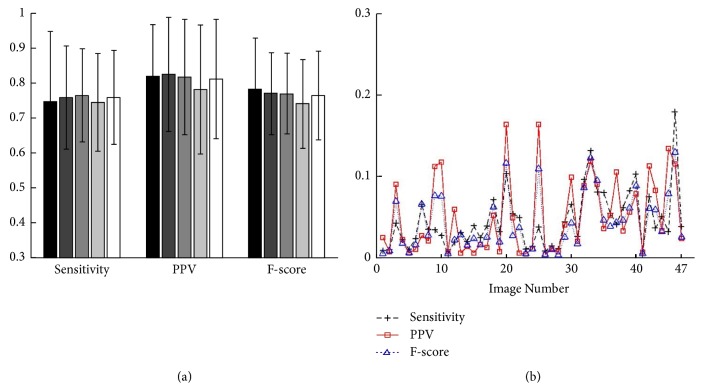
Data statistical analysis. (a) The overall mean and standard deviation of sensitivity, PPV, and F-score for all the images in the e-ophtha EX database. They are given separately for the five repeat measurements by five different operators. (b) The repeatability (standard deviation of* 5* repeat measurements by* 5* operators on each image) of sensitivity, PPV, and F-score of our algorithm on the e-ophtha EX database.

Statistical analysis showed that there was no significant difference between the five repeat measurements for the evaluation parameters (all* p>0.8*). As shown in Figure 10(a), the overall mean and SD of sensitivity, PPV, and F-score across all the images e-ophtha EX database were* 76.5%*±*15.1%*,* 82.6% *±*16.7%*, and* 76.7% *±*12.7%*. The measurement repeatability (SD of the five measurements) of sensitivity, PPV, and F-score for each individual image is shown in Figure 10(b). It ranged from* 0.3%*~*16%*, indicating that our algorithm proposed in this study for HE detection is sufficiently stable.

**Figure 11:**
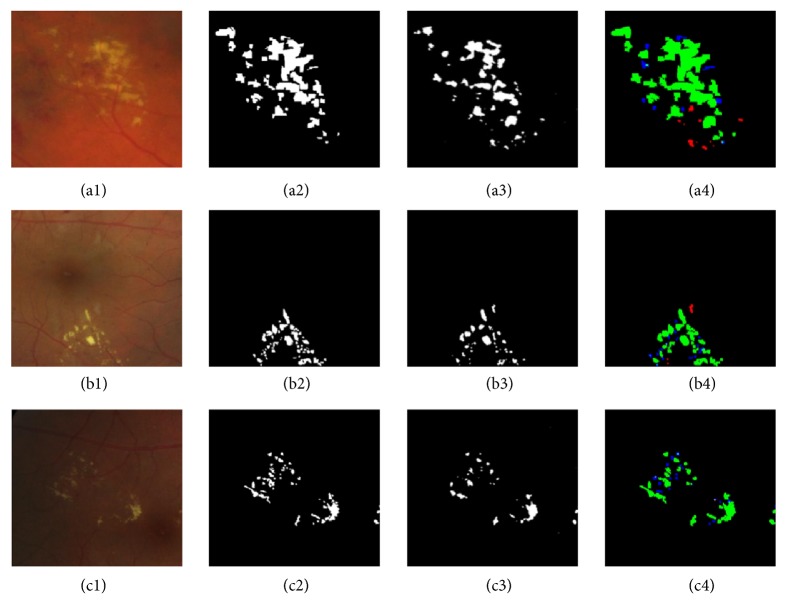
Example of pixel-level validation from three example images. (a1, b1, c1) Exudates regions cropped from the original retinal fundus image. (a2, b2, c2) The ground truth images in e-ophtha EX dataset. (a3, b3, c3) The segmented results with our algorithm. (a4, b4, c4) The results of pixel-level validation. The green, blue, red, and black pixels are the TP, FN, and FP, and TN pixels, respectively.

Table 1 also shows our algorithm achieved a higher score of PPV values in comparison with other published results also using pixel-level evaluation on the same database, indicating that our method could distinguish HE from other bright lesions more effectively. To visualize the HE detection from different retinal images, three example images are provided in Figure 11. Only the exudates regions (the left three subfigures) were cropped from the original retinal images. Figure 11(a4, b4, c4) shows the results of validation results at the pixel-level with *σ* = 0.2, where the green, red, blue, and black pixels are the TP, FN, FP, and TN pixels, respectively. It can be seen that most of the large exudates could be identified successfully. Some FPs with wrongly detected HEs could be caused by the presence of other bright lesions, such as cotton wool spots and drusens. Some small HE pixels were missed by our proposed algorithms because of their low contrasts.

### 3.2. Validation Results on DIARETDB1 Database

**Figure 12:**
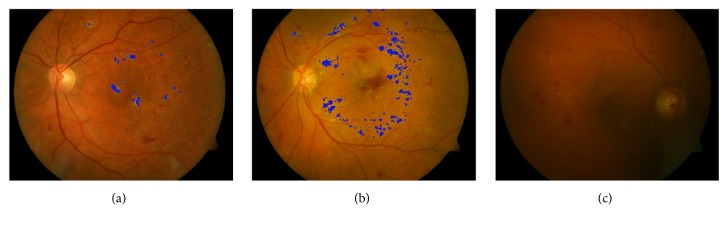
Example images showing the results of HE detection in the DIARETDB1 database. (a) HE was correctly detected. (b) Some small HE was missed in this image. (c) Failed to detect HE correctly.

Table 2 lists the overall evaluation performance of our proposed algorithm using image-level evaluation in the DIARETDB1 database. The overall mean sensitivity, specificity, and accuracy were* 97.5%*,* 97.8%,* and* 97.7%*, respectively, which compared well with other published results. Some example images from DIARETDB1 database are shown in Figure 12 to demonstrate whether an image has been correctly or wrongly detected with exudates.

## 4. Discussion and Conclusion

We have developed and evaluated an automatic retinal image processing algorithm to detect HEs using dynamic threshold, FCM and SVM. The color retinal images were segmented using dynamic threshold in combination with the global threshold, and the segmented regions were classified into two disjoint classes (exudates and nonexudates pixels) using SVM. The algorithm was tested on two publicly available databases (DIARETDB1 and e-ophtha EX database), and the evaluation results quantitatively demonstrated that our proposed algorithm is reliable in terms of repeatability and also achieved high accuracy for HE detection.

It is known that OD has similar properties with exudates in terms of color and brightness, masking or removing OD from the fundus image before further processing for HE detection is therefore important, which would improve the HE detection accuracy [10, 30, 31]. This study has presented a method for OD localization by combining the information of brightness and retinal vasculature features. Our method is inspired by Medhi et al. [23] who used a vertical Sobel mask and considered OD as the region with maximum value of edge pixels. Unlike other methods with more complicated process [29, 32], we only need to traverse the entire image twice to find the pixel with the largest gray-scale value and the most densely distributed of blood vessels, achieving fast localization of OD. Rahebi et al.'s [32] study applied the firefly algorithm and reported a success rate of* 94.38%* for OD localization in the DIARETDB1 database. Using the same database in this study, an accuracy of* 89.9%* was achieved. Although our OD detection was slightly less accurate than theirs, our method was much simpler and faster. More importantly, our method is very suitable for the application of HE detection as an intermediate step, and the relatively high accuracy was comparable with many other complex algorithms with specific aim for OD detection.

FCM has been implemented in exudates segmentation algorithms [13, 33]. Sopharak et al. [34] proposed an FCM based method to determine whether a pixel has exudates or not, but they only achieved moderately acceptable segmentation result with the sensitivity of* 80%* on DIARETDB1 database. Global threshold is commonly used for image segmentation. However, using the global information only may ignore the details from those small HEs. If the gray-scale value of background is constant, using global threshold for segmentation would achieve satisfactory results. However, in many cases, because the contrast between the object and background changes in different regions, the gray-scale value of background varies, resulting in a poor segmentation outcome. In other fields, it has been shown that using dynamic threshold in combination with the global threshold can significantly improve the segmentation results. For instance, the combined thresholds have been applied successfully to distinguish the human skin in color image and melasma image segmentation, where good segmentation results were achieved [35, 36]. The key advantage of combining the image's global information with the local details could overcome the problems associated with using local threshold alone. After employing this combined approached, the satisfactory evaluation results (*97.5%* of sensitivity on DIARETDB1 database,* 76.5%* of sensitivity on e-ophtha EX database) were achieved in this study. It is noted that only one feature (the gray-scale value of retinal images) was input into the FCM. More input features and the FCM clustering combined with the morphological technique could be also considered in future to achieve higher accuracy.

SVM classifier was selected in this study to distinguish true exudates regions from nonexudates regions. One of the key reasons is that the sample size of retinal image database used in this paper is not large enough. Using SVM was expected to have better classification result because SVM can apply the nonlinear relationship between data and features better than other classifiers [16]. Secondly, SVM can have rapid training phase [17]. Akram et al. [3] proposed a hybrid classifier as a GMM and SVM for exudates detection; however, training GMM model and finding the optimized parameters for GMM were complicated. In this study, the combined approach using FCM and SVM required less computational expenses. Only eight key features were used when compared with other algorithms with dozens of features [9, 11]. The distinguishing features of HE, in comparison with other lesions as having sharper margins and bright yellow color, enabled the most representative of eight features to be used to achieve more efficient process while maintaining the accuracy of HE extraction. Jaya et al. [37] proposed an expert decision-making system designed using a fuzzy support vector machine (FSVM) classifier to detect hard exudates. Color and texture features are extracted from the images as input to the FSVM classifier. However, using one classifier to detect HE and candidate regions of HE not extracted in advance, the computational complexity of the classifier will increase greatly, resulting in low final detection efficiency.

One limitation of our algorithm is that its performance depends on the OD detection and retinal blood vessels removal. Since the applied OD detection was quite simple in this study, the performance of our method could be further improved by improving the robustness of OD localization and blood vessel detection. Secondly, while the retinal image quality was very poor, such as the whole image is very dark with large artificial shadow (e.g., image029, image047 in DIARETDB1 database), and the contrast between HE and the background is not strong enough (e.g., image044, image052 in DIARETDB1 database), the HE detection result was poor. In addition, some big and bright cotton wool spots have been wrongly detected as HE and some small HE were ignored. In future studies, we will improve algorithms to achieve more effective detection. Furthermore, we suggest more evaluations to be carried out with the proposed algorithms on other clinically available data. Such tests could contribute to further improvements on the algorithms, resulting in more robust and more accurate detection. In summary, the satisfactory evaluation results on both retinal imaging databases demonstrated the effectiveness of employing dynamic threshold, fuzzy C-means and SVM in our proposed automatic HE detection methods, providing scientific evidence that it has potential for clinical DR diagnosis.

## Figures and Tables

**Table 1:** Overall performance comparison of our proposed algorithm with published studies for HE detection on e-ophtha EX dataset.

Methods	Sensitivity	PPV	F-score

Zhang et al. (2014) [12]	74%	72%	73%
Welfer et al. (2010) [38]	79%	55%	69%
Imani et al. (2016) [39]	80.32%	77.28%	-
Liu et al. (2017) [30]	76%	75%	76%
Kusakunniran et al. (2018) [40]	56.4%	-	-
Our proposed algorithm	76.5%	82.7%	76.7%

**Table 2:** Overall performance comparison of our proposed algorithm with published algorithms for HE detection on DIARETDB1 database.

Methods	Sensitivity	Specificity	Accuracy

Harangi et al. (2014) [11]	92%	68%	82%
Haloi et al. (2015) [9]	96.54%	93.15%	-
Imani et al. (2016) [39]	89.01%	99.93%	-
Liu et al. (2017) [30]	83%	75%	79%
Rekhi et al. (2017) [31]	91.67%	92.68%	92.13%
Fraz et al. (2017) [10]	92.42%	81.25%	87.72%
Kusakunniran et al. (2018) [40]	89.1%	99.7%	96.2%
Our proposed algorithm	97.5%	97.8%	97.7%

## Data Availability

The DIARETDB1 and the e-ophtha EX databases used to support this study are from freely available databases of retinal images at http://www.it.lut.fi/project/imageret/diaretdb1/ and http://www.eophtha.com, which have been cited. The processed data during the current study are available from the corresponding author on reasonable request.
